# Profil hématologique et nutritionnel du drépanocytaire homozygote SS âgé de 6 à 59 mois à Lubumbashi, République Démocratique du Congo

**DOI:** 10.11604/pamj.2015.21.276.6363

**Published:** 2015-08-11

**Authors:** Mick Ya Pongombo Shongo, Olivier Mukuku, Augustin Mulangu Mutombo, Toni Kasole Lubala, Paul Makinko Ilunga, Winnie Umumbu Sombodi, Stanislas Okitotsho Wembonyama, OscarNumbi Luboya

**Affiliations:** 1 Faculté de Médecine, Université de Lubumbashi, République Démocratique du Congo

**Keywords:** Drépanocytose, hémogramme, état nutritionnel, Lubumbashi, sickle cell disease, hemogram, nutritional status, Lubumbashi

## Abstract

**Introduction:**

La drépanocytose est une maladie génétique très polymorphe et la moitié des drépanocytaires homozygotes SS en Afrique meurt avant l’âge de 5 ans. Le but de cette étude est de déterminer les paramètres nutritionnels et hématologiques des jeunes drépanocytaires homozygotes (SS) congolais au cours de la phase stationnaire.

**Méthodes:**

Nous avons fait à une étude descriptive comparative de deux groupes de patients dont l'un avec 41 sujets drépanocytaires (SS) d’âge moyen de 39,1mois et l'autre, groupe contrôle, avec 82 patients avec hémoglobine AA et d’âge moyen de 35,0 mois. Nous avons eu recours à un automate ABX Micros 60 pour l’évaluation hématologique. Pour l’évaluation nutritionnel nous avons étudiés le poids, la taille, l’âge ainsi que le sexe.

**Résultats:**

Nous n'avons pas observé de différence significative entre les deux groupes par rapport à l’état nutritionnel (p > 0,05). L'hémogramme des homozygotes SS révèle une anémie chronique avec un taux moyen d'Hémoglobine à 8,33 ± 1,35g/dL. Cette anémie est normocytaire (VGM = 83,86 µm^3^), et régénérative (réticulocytes = 4,23±4,26%).

**Conclusion:**

Nos résultats rencontrent ce qui est souvent décrit dans le syndrome drépanocytaire majeur sur les homozygotes SS avec haplotype bantu.

## Introduction

Sur base des données des nations unies, 307630 bébés sont nés avec la drépanocytose en 2010 [1]. En République Démocratique du Congo, l'OMS estime à 15‰ naissances l'incidence annuelle de la forme homozygote SS et au Katanga, la prévalence attendue d'homozygotes SS est de 25‰ [2]. Une grande variabilité des données hématologiques est observée chez ces patients anémiques selon le génotype, l’âge et le sexe des patients, avec des différences selon que l'examen est réalisé au cours d'une phase stationnaire ou au cours d'une crise ou complication [3, 4]. L'absence d’études locales sur le statut hématologique et l’état nutritionnel chez ces patients drépanocytaires âgés de 6 à 59 mois en phase stationnaire constitue une difficulté pour une meilleure prise en charge. D'où, l'importance de notre étude qui nous permet d'analyser ces paramètres. L'objectif de la présente étude est d’évaluer l’état nutritionnel et les paramètres hématologiques des drépanocytaires homozygotes SS de 6 à 59 mois en stade stationnaire à Lubumbashi en RDC.

## Méthodes

Il s'agit d'une étude descriptive comparative sur les aspects nutritionnels et hématologiques de drépanocytaires homozygotes SS lushois âgés de 6 à 59 mois en phase stationnaire de la maladie. Nos recherches se sont effectuées au centre de prise en charge des drépanocytaires installé dans l'hôpital Sendwe à Lubumbashi, dans la province du Katanga au sud de la RDC. La récolte de données s'est déroulée pendant la période allant de juin 2012 à février 2013. La phase stationnaire était définie par l'absence de toute fièvre, de crise vaso-occlusive ou hémolytique. Il s'est agi d'un tirage systématique aléatoire dans une population de 201 drépanocytaires homozygotes SS âgés de 6 à 59 mois. 41 sujets (*groupe I*) ont été tirés sur base des critères suivants: sujet hémoglobinopathe SS, en phase stationnaire, figurant sur la liste de drépanocytaire recensé, reçu en consultation de routine pendant la période de l’étude et âgé de 6 à 59 mois. Le consentement verbal des parents ou tuteurs était obligatoirement sollicité avant inclusion dans l’étude. Nous avons considéré comme population de référence 82 sujets avec hémoglobine AA en bonne santé apparente et âgés de 6 à 59 mois (*groupe II*). Pour chaque sujet, les variables hématologiques (hémoglobine, hématocrite, réticulocytes, nombre de globules blancs, nombre plaquettes, nombre de globules rouges, formule leucocytaire) et nutritionnelles (poids, taille et âge) ont été recueillies sur une fiche d'enquête. Chaque sujet a bénéficié d'un prélèvement sanguin de quatre millilitres de sang total dans un tube vacuténaire à bouchon mauve contenant l'EDTA.K_3_ après ponction veineuse pour la réalisation d'un hémogramme complet sur un automate (ABX Micros 60) et de l’électrophorèse de l'hémoglobine à pH alcalin sur un équipement à électrophorèse Sebia. Le logiciel Epi info 7.0.8.3 a été utilisé pour la gestion et l'analyse des données et le logiciel ENA for Smart a été utilisé pour l’évaluation nutritionnelle. Nous avons utilisé les tests de statistiques descriptives qui nous ont permis de calculer la moyenne et l’écart-type. Et comme test de statistique différentiel, le t de Student a été utilisé pour la comparaison des moyennes entre les deux groupes. Considérations éthiques: la recherche pour réaliser ce travail a été autorisée par le comité d’éthique de l'Université de Lubumbashi. Un consentement libre et éclairé (verbal ou écrit) de toutes les personnes impliquées dans cette étude a été obtenu au préalable.

## Résultats

### Evaluation de l’état nutritionnel (Tableau 1)

Le z-score P/A moyen est de -0,18±1,01 ET (extrêmes: -2,42 et 1,73) et la proportion des insuffisants pondéraux est de 4,9% dans le groupe I alors qu'ils sont respectivement de 0,06±1,12 ET (extrêmes: -4,18 et 2,77) et de 2,4% dans le groupe II (p > 0,05). Pour ce qui est de l’évaluation de la croissance staturale, le z-score T/A moyen est de -0,71±1,49 (extrêmes: -3,06 et 1,49) et la proportion des malnutris chroniques est de 19,5% dans le groupe I alors qu'ils sont respectivement de -0,31±1,6 ET (extrêmes: -5,00 et 3,80) et de 12,2% dans le groupe II (p > 0,05). Quant au z-score P/T, la moyenne dans le groupe I est de 0,27±1,51 (extrêmes: -4,10 et 3,23) et la proportion des malnutris aigus est de 9,8% alors que dans le groupe II elles sont respectivement de 0,29±1,23 ET (extrêmes: -3,27 et 2,61) et de 3,7% (p > 0,05).

**Tableau 1:** Evaluation de l’état nutritionnel

Paramètre	Groupe I (n = 41)	Groupe II (n = 82)	p
**Z-score P/A**			
<-2 ET	2 (4,9%)	2 (2,4%)	0,3335
≥-2 ET	39 (95,1%)	80 (97,6%)	
Moyenne	-0,18±1,00	0,07±1,11	0,2203
**Z-score P/T**			
<-2 ET	4 (9,8%)	3 (3,7%)	0,3354
≥-2 ET	37 (90,2%)	79 (96,3%)	
Moyenne	0,28±1,51	0,29±1,23	0,9618
**Z-score T/A**			
<-2 ET	8 (19,5%)	10 (12,2%)	0,4169
≥-2 ET	33 (80,5%)	72 (87,8%)	
Moyenne	-0,71±1,49	-0,31±1,66	0,1925

### Paramètres hématologiques (Tableau 2)

Concernant les hématies, les moyennes sont respectivement de 3,1±0,5 tera/L (extrêmes: 2,3 et 3,3 tera/L) et 3,5±0,6 tera/L (extrêmes: 2,8 à 5,3 tera/L) dans les groupes I et II. L'analyse statistique montre une différence significative quant au taux de GR entre les deux groupes (*t*=13,39 et p < 0,0001). Le taux d'hémoglobine a varié entre 5,6 et 10,5 g/dL autour d'une moyenne de 8,3±1,4 g/dL dans le groupe I et entre 7,6 et 12,9 g/dL autour d'une moyenne de 11,6±1,7 g/dL dans le groupe II. La comparaison de ces deux moyennes montre une différence statistiquement significative (*t*=114,05; p < 0,0001). En ce qui concerne le pourcentage d'hématocrite, il a varié entre 16,0% et 34,0% autour d'une moyenne de 25,7±4,3% dans le groupe I et entre 26,0% et 45,0% autour d'une moyenne de 35,3±4,4% dans le groupe II. En comparant ces deux moyennes, l'analyse donne une différence statistiquement significative (*t*=131,98; p < 0,0001). Concernant le pourcentage de réticulocytes a varié entre 1,7 et 5,2% autour d'une moyenne de 2,9±0,7% dans le groupe I contre 1,7% et 5,6% autour d'une moyenne 3,2±0,8% dans le groupe II. La comparaison de ces moyennes ne donne pas une différence statistique significative (*t*=1,51; p = 0,2209). Quant au taux de globules blancs, la moyenne est de 9,9±2,4 giga/L (extrêmes: 10,5 et 17,5 giga/L) dans le groupe I alors qu'elle est de 94,8±2,7 giga/L (extrêmes: 7,50 à 9,70 giga/L) dans le groupe II. L'analyse statistique montre une différence significative quant à la comparaison de ces deux moyennes (*t*=11,29; p = 0,0010). En outre, nous n'avons pas observé des différences statistiquement significatives en fonction des éléments de la formule leucocytaire: monocytes: moyennes respectivement de 3,7±3,3% et 3,2±3,0% dans les groupes I et II (p = 0,3813); neutrophiles: moyennes respectivement de 39,9±12,6% et 42,3±13,2% dans les groupes I et II (p = 0,3411); lymphocytes: moyennes respectivement de 58,6±15,9% et 52,1±16,3% dans les groupes I et II (p = 0,6233). Le taux de plaquettes s’étend de 167,0 à 311,0 giga/L dans le groupe I avec une moyenne de 247,5 ± 44,6 giga/L contre 156,0 à 311,0 giga/L dans le groupe II avec une moyenne de 210,3±43,5 giga/L). La comparaison de deux moyennes ne montre pas de différence significative (*t*= 0,73; p = 0,3960).

**Tableau 2:** Paramètres hématologiques de drépanocytaires et du groupe de référence

Paramètre	Groupe I (n = 41)	Groupe II (n = 82)	p
Hématies (tera/L)	3,14 ± 0,49	3,54 ± 0,60	0,0003
Hémoglobine (g/dL)	8,33 ± 1,35	11,64 ± 1,73	<0,0001
Hématocrite (%)	25,68 ± 4,28	35,25 ±4,39	<0,0001
VGM (m^3^)	83,86 ± 19,95	100,67 ± 11,69	<0,0001
HCM (pg)	27,17 ± 6,18	33,14 ± 4,00	<0,0001
CCMH (%)	32,57 ± 2,36	32,98 ± 2,33	0,3522
Réticulocytes (%)	4,23 ± 4,26	3,15 ± 0,76	0,0284
Plaquettes (giga/L)	247,48 ± 44,57	210,34 ± 43,51	0,3960
Leucocytes (giga/L)	11,23 ± 2,69	94,79 ± 2,70	<0,001
Neutrophiles (%)	39,92 ± 12,63	42,30 ± 13,1897	0,3411
Monocytes (%)	3,68 ± 3,27	3,15 ±3,04	0,38129
Lymphocytes (%)	53,60 ±15,89	52,08 ± 16,32	0,6233

VGM: volume globulaire moyen; CCMH: concentration corpusculaire moyenne en hémoglobine

## Discussion

La comparaison des indices anthropométriques de l’évaluation de l’état nutritionnel entre les enfants drépanocytaires et ceux avec hémoglobine AA n'a montré aucune différence significative. Plusieurs auteurs reconnaissent qu'un pauvre état nutritionnel est une des caractéristiques souvent associées à la drépanocytose, en particulier avec la forme homozygote SS [5–9]. Il faut signaler cependant que le retard de croissance staturo pondéral est plus marqué à la puberté chez l'enfant drépanocytaire [10, 11]. Pour déterminer l′influence d'une hémoglobinopathie sur la croissance et le développement, Platt a examiné la taille et le poids de 2115 patients âgés de 2 à 25 ans avec syndrome drépanocytaire majeur et a constaté que les courbes sont significativement différentes des normes publiées par l'OMS (p < 0,001) et que les sujets homozygotes SS étaient toujours plus petits et moins gros (p < 0,001) [10, 12]. Wolman a observé que les patients souffrant de la β-thalassémie et traités par une thérapie transfusionnelle intensive apparaissent en meilleure santé et leur croissance est plus proche de la normale que ceux transfusés uniquement lorsque l'hémoglobine tombait à des niveaux faibles [13]. Des observations similaires ont été faites par Kattamis et coll [14] qui ont conclu que les transfusions intensives constituent le traitement de choix si la croissance normale doit être assurée. Cependant, il est difficile de savoir si l′insuffisance de nutriments est due à une mauvaise alimentation ou une mauvaise absorption ou une utilisation défectueuse des métabolites par l'individu [15]. L'hémogramme du sujet drépanocytaire homozygote SS dans notre étude montre au cours des phases stationnaires une anémie constante, d'intensité variable (8,33±1,35 g/dL), normocytaire (VGM moyen 83,86 µm^3^), régénérative avec une moyenne de réticulocytes à 4,23%. La différence est significative par rapport au groupe de référence (p < 0,05). Cette différence observée entre ces moyennes dans les deux groupes sont en accord avec les études de Nacoulma, Tshilolo et Omoti [16–18]. Concernant les hématies, les moyennes sont statiquement basses dans le groupe drépanocytaire SS par rapport au groupe de référence (p < 0,05). Les patients drépanocytaires même en dehors des crises ont continuellement une hémolyse des globules rouges avec un taux de survie court des érythrocytes entre 12 et 14 jours [19]. Il est vrai que les drépanocytaires SS de notre série étaient tous sous acide folique, mais il est difficile d'attester l'observance du traitement car celui-ci était pris à domicile. Cependant, Bazuaye observe une différence du taux d'hémoglobine chez le drépanocytaire sous acide folique déjà après 14 jours de traitement [20]. Chez les sujets drépanocytaires homozygotes, la chute de l'hémoglobine f'tale (HbF) après la naissance est quelque peu retardée et le taux se stabilise vers l’âge de 5-6 ans. Un taux élevé d'HbF laisse espérer une bonne évolution de la maladie [17]. C'est surement ce qui est observé dans notre étude où l’âge s’étend de 6 à 59 mois.

La moyenne de leucocytes dans le groupe drépanocytaire est significativement plus élevés que celle du groupe de référence (p = 0,001). La drépanocytose est en effet une maladie inflammatoire dont l′un des marqueurs est la leucocytose [21]. L′augmentation du nombre et l′activation des leucocytes sont des médiateurs importants de l′inflammation dans la drépanocytose. Les leucocytes peuvent adhérer les uns aux autres, aux érythrocytes falciformés ou non, aux plaquettes et à l′endothélium vasculaire [17]. Il faut reconnaitre que dans les hémolyses aigues, la forte régénération médullaire est responsable d'une érythroblastose à l'origine d'une fausse hyperleucocytose; puisque les érythroblastes du fait de leur noyau sont comptés comme des leucocytes par les automates. En effet, cette leucocytose non corrigée est plus importante chez les SS [16]. Un taux élevé de leucocytes est associé à un risque plus élevé de décès précoce et semble être un facteur prédictif de mauvais pronostic [22]. Par exemple le taux élevé de la L-sélectine (CD62) de lymphocyte est corrélée à la survenue de l′accident vasculaire cérébral tandis que celui de ‘2 intégrine (CD18) de neutrophile prédispose à la néphropathie drépanocytaire [23]. Nous avons observé une formule à prédominance lymphocytaire dans les deux groupes avec une légère supériorité dans le groupe drépanocytaire avec 58,6 ± 15,9% contre 52,1 ± 16,3% dans le groupe de référence. Cette différence n'est pas statistiquement significative (p > 0,05). Le nombre moyen de plaquettes à la phase stationnaire (247,87 109/L) dans notre série est inférieur aux moyennes (320-327 109/L) rapportées dans d'autres études [24, 25]. Mais il est était plus élevé que dans le groupe de référence (210,34 ± 43,51 109/L). Ce résultat est en accord avec les études de Jaffe à Ontario au Canada et Osaghae à Benin city au Nigeria, qui montrent que la numération plaquettaire est supérieure en cas de drépanocytose comparativement à la population de référence [24, 25]. Toutefois, nous n'avons pas observé de différence significative (p > 0,05). Les plaquettes activées sécrètent la thrombospondine (TSP) impliquée dans le pontage GR-endothélium et participent à l’état d'hypercoagulabilité de la maladie drépanocytaire contribuant ainsi à la survenue des crises [17].

## Conclusion

Les résultats de notre étude rencontrent ce qui est souvent décrit dans le syndrome drépanocytaire majeur sur les homozygotes SS avec haplotype bantu.
